# A Hybrid Approach of Stepwise Regression, Logistic Regression, Support Vector Machine, and Decision Tree for Forecasting Fraudulent Financial Statements

**DOI:** 10.1155/2014/968712

**Published:** 2014-09-11

**Authors:** Suduan Chen, Yeong-Jia James Goo, Zone-De Shen

**Affiliations:** ^1^Department of Accounting Information, National Taipei University of Business, 321 Jinan Road, Section 1, Taipei 10051, Taiwan; ^2^Department of Business Administration, National Taipei University, No. 67, Section 3, Ming-shen East Road, Taipei 10478, Taiwan

## Abstract

As the fraudulent financial statement of an enterprise is increasingly serious with each passing day, establishing a valid forecasting fraudulent financial statement model of an enterprise has become an important question for academic research and financial practice. After screening the important variables using the stepwise regression, the study also matches the logistic regression, support vector machine, and decision tree to construct the classification models to make a comparison. The study adopts financial and nonfinancial variables to assist in establishment of the forecasting fraudulent financial statement model. Research objects are the companies to which the fraudulent and nonfraudulent financial statement happened between years 1998 to 2012. The findings are that financial and nonfinancial information are effectively used to distinguish the fraudulent financial statement, and decision tree C5.0 has the best classification effect 85.71%.

## 1. Introduction

The financial statement is the main basis of decision-making by investors, creditors, and other accounting information demanders and concurrently also the concrete expression of management performance, financial condition, and possessing social responsibility of the listed and OTC companies, but the fraudulent financial statement (FFS) has the trend of becoming increasingly serious in recent years [1–8].

This behavior not only makes the investing public subject to vast amount of loss but also, more seriously, influences the capital market order. Because the fraudulent case is increasingly serious with each passing day, the United States Congress passed Sarbanes-Oxley Act in 2002 and mainly hope by which to improve the accuracy and reliability of the financial statement of a company and disclosure to make the auditors able to forecast the omen of the FFS before the FFS of an enterprise occurs. When one checks corporations' financial statements due to fraud which led to a significant misstatement, there are fairly strict norms for audit staff in Taiwan [9].

The FFS can be regarded as a typical classification problem [10]. The classification problem carries out a computation mainly in light of the variable attribute numerical value of some given classification data to acquire the relevant classification rule of every classification and bring the unknown classification data into the rule to acquire the final classification result. Many authors apply the logistic regression to make a fraudulent classification and acquire the result in the FFS issue in the past [3, 6, 7, 11–13].

Data mining is an analytical tool used to handle a complicated data analysis. It discovers previously unknown information from mass data and aims for data to make an induction from the structured model as reference amount in making a decision with many different functions, such as classification, association, clustering, and forecasting [4, 5, 8, 14]. “Classification” function is used the most often therein, and its result can serve as the decision basis and prediction. However, whether every application of data mining in the FFS is superior to the traditional classification model is controversial.

The purpose of this study is to expect that a better method of forecasting fraudulent financial statement can be presented to forecast the omen of the fraudulent financial statement and to reduce damage to the investors and auditors. The study will adopt the logistic regression and the support vector machine (SVM) as well as the decision tree (DT) C50 in data mining as the basis and match the stepwise regression to separately establish classification model to make a comparison. In conclusion, the study first aims at the “fraudulent financial statement” issue to make an arrangement for and carry out an exploration of relevant literature to ensure the research variable and sample adopted by the study. We then take the logistic regression, SVM, and DT C5.0 as the bases to establish the FFS classification model. Finally, we present the conclusions and suggestions of the study.

## 2. Literature Review

### 2.1. Fraudulent Definition

The FFS is a kind of intentional or illegal behavior, the result of which directly causes the seriously misleading financial statement or financial disclosure [2, 15]. Pursuant to the provision of SAS NO.99, a kind of fraudulent pattern is dishonest financial report, and it means a kind of intentional erroneous narration, neglecting amount or disclosure, which makes the misunderstood financial statement [6].

### 2.2. Research Method

The classification problem carries out a computation mainly in light of the variable attribute numerical value of some given classification data to acquire the relevant classification rule of every classification and bring the unknown classification data into the rule to acquire the final classification result. Many authors apply the logistic regression to make a fraudulent classification in the FFS issue in the past [3, 11, 12, 15–17]. However, the traditional statistic method has limitation of having to accord with specific assumption in data.

As a result, the machine learning way which does not require any statistic assumption about data portfolio rises abruptly. Many scholars recently try to adopt the machine learning way as the classification machine to conduct a research. The empirical result also points out that it possesses an excellent classification effect. Chen et al. [13] applied the neural network and SVM to forecast network invasion, and the research result indicates that the SVM has excellent classification ability. Huang et al. [18] applied the neural network and SVM to explore the classification model of credit evaluation. Shin et al. [19] conducted a relevant research of bankruptcy prediction. Yeh et al. [4] apply it in prediction of enterprise failure. On the other hand, Kotsiantis et al. [3] and Kirkos et al. [10] apply DT C5.0 in the relevant research to acquire the excellent classification result. Thus, the study will adopt the foresaid logistic regression, SVM, and DT C5.0 as the classifier construction classification model.

### 2.3. Variable Selection

As for variable selection via relevant literature exploration, some authors adopt the financial variable as the research variable [3, 10], others adopt the nonfinancial variable as the research variable [12, 16, 17], and still others adopt both the financial variable and nonfinancial variable as the research variable [15, 20].

Because financial statement data often have cheating suspicion, if we purely consider the financial variables, the possibility of erroneous classification may increase. Therefore, the study not only adopts the financial variable as the research variable, but also adds the nonfinancial variable to construct the fraudulent financial prediction model.

## 3. Methodology

The purpose of this study is to present a two-stage research model which integrates the financial variable and nonfinancial variable to establish the fraudulent early warning model of an enterprise. The procedure of the study is to aim at the data to make a stepwise regression analysis, to acquire the result of the important variable of the TTF after screening, and then to take such variable as the input variable of the logistic regression and SVM. Finally, the study makes a comparison and an analysis to acquire a better FFS classification result.

### 3.1. Stepwise Regression

The study selects a variable of the maximum classification ability in accordance with forward selection and incorporates the predictor into the model by stepwise increase. During each process, *P* value of the statistic test is used to screen the variables. If *P* value is less than or equal to 0.05, then the variable enters the regression model, and the selected variable is the independent variable of the regression model.

### 3.2. Logistic Regression

The logistic regression resembles the linear regression, while the response variable and explanatory variable of the general linear regression are usually the continuous variable, but the response variable explored by the logistic regression is the discrete variable; that is, it handles the qualitative variable of the two-dimensional independent variable problem (e.g., yes or no and success or failure). The model utilizes cumulative probability density function to convert real number value of the explanatory variable to probability value between 0 and 1. The elementary assumption is different from the analytic assumption of another multivariate analysis. The influence of the explanatory variable on the response variable is to fluctuate in the index form, which means that the logistic regression does not need to conform to the normal distribution assumption. In other words, it can handle the population of the nonnormal distribution and the problem of the nonlinear model and the nonmeasuring variable.

The general logistic regression model is as follows:

(1) $$\begin{array}{c} Y^{*}=\beta x+\varepsilon \\ Y=\left(\begin{array}{cc} 1: & Y^{*}>0\\ 0: & Y^{*}\leq 0 \end{array}\right), \end{array}$$

where *Y*: response variable of actual observation, *Y* = 1: a financial crisis event occurs, *Y* = 0: no financial crisis event occurs, Y*: latent variable without observation, *x*: matrix of explanatory variable, *β*: matrix of explanatory variable parameter, and *ε*: error of explanatory variable.

### 3.3. Support Vector Machine (SVM)

The operation model of the SVM projects the initial input vector to eigenspace of the high dimension with linear and nonlinear core function and utilizes the separating hyperplane to distinguish two or many materials of different classes. The SVM utilizes the hyperplane classifier to classify the materials.

#### 3.3.1. Linear Divisibility

When the plain formed by the training sample data is linear, which consider the training vector: *x*_*i*_ = (*x*_*i*_^(1)^,…, *x*_*i*_^*n*^) ∈ *R* ^*n*^ belongs to two classes *y*_*i*_ ∈ {−1, +1}. In order to definitely distinguish the training vector class, it is necessary to find out the optimal partition hyperplane able to separate the materials.

If the hyperplane *w* · *x* + *b* can separate the training sample, it is shown as

(2) $$\begin{array}{c} w\cdot x_{i}+b>0,\;\text{if}\;y_{i}=1 \end{array}$$

(3) $$\begin{array}{c} w\cdot x_{i}+b<0,\;\text{if}\;y_{i}=-1. \end{array}$$

Adjust *w* and *b* properly; (2) and (3) can be rewritten as

(4) $$\begin{array}{c} w\cdot x_{i}+b\geq 1,\;\text{if}\;y_{i}=1\\ w\cdot x_{i}+b\leq -1,\;\;\text{if}\;y_{i}=-1. \end{array}$$

or as

(5) $$\begin{array}{c} y_{i}\left(w\cdot x_{i}+b\right)\geq 1,\;\forall i\in \left\{1,\ldots ,n\right\}. \end{array}$$

Pursuant to the statistics theory, the best interface not only separates two classes of samples correctly, but also maximizes the classification margin. The class margin of the interface *w* · *x* + *b* is shown as

(6) $$\begin{array}{c} d\left(w,b\right)=\underset{\left\{x_{i}\;|\;y_{i}=1\right\}}{\min}\frac{w\cdot x_{i}+b}{\left|w\right|}-\underset{\left\{x_{i}\;|\;y_{i}=-1\right\}}{\max}\frac{w\cdot x_{i}+b}{\left|w\right|}. \end{array}$$

Equation (7) can be acquired from (4):

(7) $$\begin{array}{c} d\left(w,b\right)=\frac{1}{\left|w\right|}-\frac{-1}{\left|w\right|}=\frac{2}{\left|w\right|}. \end{array}$$

So the problem of the maximization class margin *d*(*w*, *b*) transforms to minimization |*w*|^2^/2 under constraint condition (5). Pursuant to Lagrange relaxation, the foresaid problem must accord with the hypothesis of (8) and (9). In the foresaid condition, the minimization is shown as (10):

(8) $$\begin{array}{c} \alpha_{i}\geq 0 \end{array}$$

(9) $$\begin{array}{c} \underset{i}{\sum }\alpha_{i}y_{i}=0 \end{array}$$

(10) $$\begin{array}{c} \underset{i}{\sum }\alpha_{i}-\frac{1}{2}\underset{i,j}{\sum }\alpha_{i}\alpha_{j}y_{i}y_{j}x_{i}\cdot x_{j},\;i=1,\ldots ,n. \end{array}$$

Every *α*_*i*_ corresponds to a training sample *x*_*i*_, and the training sample of its corresponding *α*_*i*_ > 0 is called the support vector. Classification function acquired finally is shown as

(11) f(x)=sgn⁡(w·xi+b)=sgn⁡(∑i=1Nsαiyixi·x+b),

where *N*_*s*_ is the number of the support vector.

#### 3.3.2. Linear Indivisibility

If the training sample is linearly indivisible, (4) can be rewritten as

(12) $$\begin{array}{c} w\cdot x_{i}+b\geq 1-\xi_{i},\;\text{if}\;y_{i}=1\\ w\cdot x_{i}+b\geq \xi_{i}-1,\;\text{if}\;y_{i}=-1. \end{array}$$

where *ξ*_*i*_ ≥ 0, *i* = 1,…, *n*.

If *x*_*i*_ is classified mistakenly, then *ξ*_*i*_ > 1. Thus, the mistaken classification is less than ∑_*i*_ *ξ*_*i*_. Add a given parameter value in the objective function. Consider reasonably the maximum class margin and the minimum mistaken class sample; that is, seeking the minimum of |*w* ^2^|/2 + *C*(∑_*i*_ *ξ*_*i*_) can acquire the SVM under linear indivisibility. Pursuant to Lagrange relaxation, the foresaid problem must accord with the hypothesis of (13) and (14). In the foresaid condition, the minimization is shown as (15):

(13) $$\begin{array}{c} 0\leq \alpha_{i}\leq C \end{array}$$

(14) $$\begin{array}{c} \underset{i}{\sum }\alpha_{i}y_{i}=0 \end{array}$$

(15) $$\begin{array}{c} \underset{i}{\sum }\alpha_{i}-\frac{1}{2}\underset{i,j}{\sum }\alpha_{i}\alpha_{j}y_{i}y_{j}x_{i}\cdot x_{j},\;\;i=1,2,3,\ldots ,n. \end{array}$$

### 3.4. Decision Tree (DT)

The Decision Tree (DT) is the simplest in the inductive learning method [21]. It belongs to the data mining tool and can handle the continuous and noncontinuous variable. It establishes the tree structure diagram mainly by the given classification fact and induces some principles therein. The principles are mutually exclusive, and the DT generated can also make an out-of-sample prediction. The DT algorithms used most frequently include CART, CHAID, and C5.0 [22]. C5.0 [23] improves from ID3 [23]. Thanks to ID3 use limitation, it cannot handle the continuous numerical value materials; thus, Quinlan conducts a research for improvement, and C5.0 is developed to handle the continuous and the noncontinuous numerical value.

The DT C5.0 is mainly separated into two parts. The first part is classification criterion, which is calculated pursuant to the gain ratio. Construct the DT completely as shown in (2). Information gained in (16) is used to calculate the pretest and posttest gain of the data set and is defined as “pretest information” minus “postinformation” from (17). The entropy in (16) is used to calculate impurity, which is called randomness. In other words, it is used to calculate randomness of the data set. When randomness in the data set reaches the most disorderly state, the value will be 1.

Therefore, the less random the posttest data set is, the larger the information gain is calculated, and the more favorable it is for DT construction:

(16) $$\begin{array}{c} \text{Gain}\;\text{Ratio}\left(S,A\right)=\frac{\text{Information}\;n\;\text{Gain}\;\left(S,A\right)}{\text{Entropy}\left(S,A\right)} \end{array}$$

(17) $$\begin{array}{c} \text{Gain}\left(S,A\right)=\text{Entropy}\left(S\right)-\underset{v\;\in \text{values}\left(A\right)}{\sum }\frac{\left|S_{v}\right|}{\left|S\right|}\text{Entropy}\left(S_{v}\right). \end{array}$$

The second part is pruning criterion. Pursuant to the error based pruning (EBP), the DT is properly pruned to enhance the correct ratio of classification. EBP is evolved from the pessimistic error pruning (PEP), and such two pruning methods are presented by Quinlan. The main concept of the EBP is to make a judgment using the error ratio, calculate the error ratio of every node, and further judge the node which results in rise of the error ratio of the overall DT. Finally, this node is pruned properly to further enhance the correct ratio of the DT.

### 3.5. Definition of Type I Error and Type II Error

In order to establish the valid forecasting fraudulent financial statement, it is considerably important to measure type I type II errors of the study. Type I error is to mistakenly judge the normal financial statement company as the FFS company. This judgment does not cause investors' damage, but it carries out an erroneous audit opinion for being too conservative and further influences credit of the company audited. Type II error is that the FFS enterprise is mistaken for the normal enterprise. This classification error leads to auditing failure, auditors' investment loss, or investors' erroneous judgment.

## 4. Empirical Analysis

### 4.1. Data Collection and Variables

The research samples are the FFS enterprises from the years 1998 to 2012. 66 enterprises are selected from the listed and OTC companies of the Taiwan Economic Journal Data Bank (TEJ). The 1 by 1 pair way is adopted to match 66 normal enterprises, so there are 132 enterprises in total as research samples.

As for selection of the research variables, the study altogether selects 29 variables, including 24 financial variables and 5 nonfinancial variables (see appendix).

For consideration of the number of samples, to avoid having too few samples of the test group and to improve test accuracy, we propose to utilize 50% of the sample materials as the train sample to establish the regression classification model. The remaining 50% of the sample materials serve as the test sample to test validity of the classification model established.

**Figure 1:**
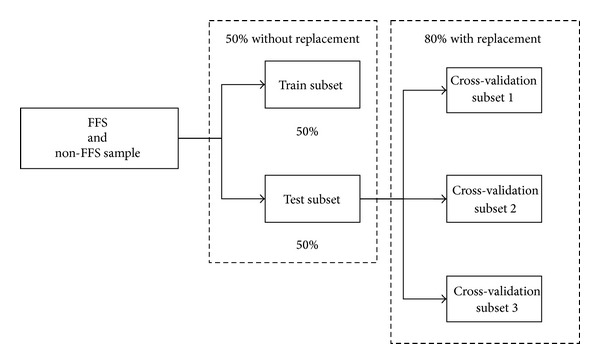
Train and test subsets design.

In addition, to test the stability of the proposed research model, this study randomly selects three groups at a ratio of 80% from the test data as the test sample for cross-validation. The compartment and sampling of data in this research are shown in Figure 1.

### 4.2. Model Development

**Figure 2:**
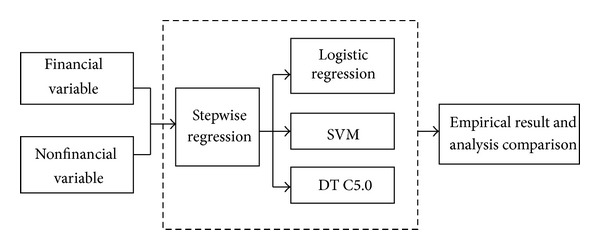
Research model.

To begin with, the study aims for the financial and nonfinancial variable to screen using the stepwise regression screening method. The variables screened serve as the input variable of the logistic regression and SVM. Next, the study aims at every method to carry out the model training and test. Finally, the study compares the merit and demerit of the classification correct ratio and gives the relevant suggestions for the analytic result. The model construction is divided into three parts. The first part is the variable screening way; the second part is the classification way; the third part compares the test results of two kinds of classification models. The research process of the study is shown as Figure 2.

### 4.3. Important Variable Screening

While constructing the classification model, there may be quite many variables, but not every variable is important. Therefore, the variables of no account need to be eliminated to construct a simpler classification model. There are quite many variable screening ways, among which the stepwise regression variable screening method is used most frequently [24].

Therefore, the study adopts the suggestions of Pudil et al. [24] to screen the variables using the stepwise regression by which to retain the research variables with more influence. The input variables of the study are screened via the stepwise regression to acquire the results as shown in Table 1, including 7 financial variables and 1 nonfinancial variable. Subsequently, the study takes these 8 variables as the new input variables to construct the classification model.

### 4.4. Classification Model

The prediction accuracy of the three types of models using the train datasets is displayed in Table 2.

As shown in Table 2, C5.0 has the best performance in the establishment of the prediction model and its accuracy rate is 93.94%. The traditional logistic model is the second best. The accuracy rate of the SVM model, at 78.79%, is the lowest of the three. The cross-validation results of the proposed three prediction models are shown in Tables 3 to 5.

#### 4.4.1. Decision Tree (DT)

The study constructs the DT C5.0 model, sets EBP at *α* = 5%, and adopt the binary partition principle to obtain the optimal spanning tree. The prediction results of the DT C5.0 classification model are shown as Table 3.

On average, 25 of the 28 non-FFS materials are correctly classified in the non-FFS, and three of them are incorrectly classified in the FFS. The type I error is 10.71%. On the other hand, 23 of the 28 FFS materials are correctly classified, and the remaining five FFS materials are incorrectly classified in the non-FFS. The type II error is 17.85%.

#### 4.4.2. Logistic Regression

Table 4 is the empirical results of the logistic classification model, which shows that 25 of 28 non-FFS materials are correctly classified and that three of them are incorrectly classified in the FFS. The overall type I error is 9.52%. In addition, 20 of the 28 FFS materials are correctly classified, and the remaining eight FFS materials are incorrectly classified in the non-FFS. The type II error is 28.57%.

#### 4.4.3. Support Vector Machine (SVM)

The operation core is set at RBF when the study constructs the SVM model. As for the parameter, the C search scope is set at 2^−10^ to 2^10^, and *γ* is set at 0.1. The SVM classification results are shown as Table 5.

In this part, 26 of the 28 non-FFS materials are correctly classified, and two of them are incorrectly classified in the FFS. The type I error is 7.14%. In addition, 14 of the 28 FFS materials are correctly classified, and the remaining 14 FFS materials are incorrectly classified in the non-FFS. The type II error is 48.81%.

#### 4.4.4. Comprehensive Comparison and Analysis

Kirkos et al. [10] pointed out that the merit and demerit of the evaluation model must also consider the type I error and type II error. The type I error means to classify the nonfraudulent companies into the fraudulent companies. Occurrence of these two type errors results from the auditing failure of the auditors. Type II error means that the auditors classify the fraudulent companies into the nonfraudulent companies. Both types of error would cause different loss costs, and the auditors must avoid occurrence of these two errors. Comparing the results of these three models, we conclude that the classification ability of the DT C5.0 is the best, the next is the logistic regression, and the last is the SVM. The classification correct ratios of three kinds of model are summarized as shown in Table 6.

The comparison shows that, although the logistic classification model performs the best for type I errors, the DT C5.0 possesses the best classification effect, both for type II errors and the hit ratio. The correct classification ratio is 85.71%, followed by 80.95% for the logistic model, and 72.02% for the SVM model.

Unlike general studies using type I errors to judge the performance of prediction models, FFS studies use type II errors to determine the performance of prediction models. For the sake of prudence, we conduct the statistical test of type II errors in the abovementioned cross-validation results to confirm whether the differences in between models are significantly other than 0. The analysis results are shown in Table 7, which shows that the *t*-values of the prediction model type II error differences are −5.201 (C5.0—Logistic); −16.958 (Logistic—SVM); and 9.823 (SVM— C5.0), respectively, and all of them reach the significance level.

## 5. Conclusion and Suggestion

As the fraudulent financial statement (FFS) increases on the trot in recent years, the auditing failure risk of the auditors also rises thereby. Therefore, many researches focus on developing a good classification model to reduce the relevant risk. In the past, the accuracy of forecasting FFS purely using regression analysis has been relatively low. Many scholars have pointed out that prediction by data mining can improve the accuracy rate. Thus, this study adopts stepwise regression to screen the important factors of financial and nonfinancial variables. Meanwhile, it combines the above with data mining techniques to establish a more accurate FFS forecast model.

A total of eight critical variables are screened via the stepwise regression analysis, including two parts: financial variables (accounts receivables/total assets, inventory/current assets, interest protection multiples, cash flow ratio, accounts payable turnover, operation profit/last year operation profit > 1.1) and nonfinancial variables (pledge ratio of shares of the directors and supervisors).

The financial variables include operating capabilities, profitability index, debt solvency ability index, and financial structure. The nonfinancial variables include relevant variables of stock rights and scale of an enterprise's directors and supervisors. The results indicate that when auditors investigate FFS, they must focus on the alert provided by the nonfinancial information as well as the financial information.

In the classification model, the study adopts the logistic regression of the traditional classification method and the DT C5.0 and SVM of data mining to construct the classification model. The empirical result indicates that the SVM model performs the best in the type I error after comparison, and the DT C5.0 has the best classification performance in the type II error and overall classification correct ratio.

One of the research purposes is to anticipate accommodating the auditors with another assistant auditing tool besides the traditional analysis method, but the research about the forecasting FFS is not sufficient. Therefore, the subsequent researchers can also adopt other methods to forecast the FFS to provide a better reference. In addition, future researchers can also try to adopt different variable screening methods to enhance the classification correct ratio of the method. As for the variable, some nonfinancial variables are difficult to measure, and material acquisition is difficult, so the study does not incorporate them. Finally, as for the sample, the study focuses on the FFS scope research, and a certain number of the FFSs may not be found. Therefore, the pair companies can also be the FFS companies in the coming year, which can influence the accuracy of the study. The findings of this study can provide a reference to auditors, certified public accountants (CPAs), securities analysts, company managers, and future academic studies.

## Figures and Tables

**Table 1:** Results of stepwise regression variable screening.

Variable code	Variable classification	Variable description	Pr > ChiSq
*X*1	Financial	Accounts receivables/total assets	0.2401
*X*3	Financial	Inventory/current assets	0.0339
*X*10	Financial	Interest protection multiples	0.0694
*X*13	Financial	Debt ratio	0.0294
*X*15	Financial	Cash flow ratio	0.0025
*X*17	Financial	Accounts payable turnover	0.0295
*X*24	Financial	Operation profit/last year operation profit >1.1	0.0267
*X*29	Nonfinancial	Pledge ratio of shares of the directors and supervisors	0.0473

**Table 2:** Hit ratio of three models using the train datasets.

Research model	C5.0	Logistic	SVM
Hit ratio	93.94%	83.33%	78.79%

**Table 3:** C5.0 cross-validation results.

C5.0 model			Predict value		Hit ratio	Type I error	Type II error
			Non-FFS	FFS			
Actual value	CV1	Non-FFS	25	3	83.93%	10.71%	21.42%
		FFS	6	22			
	CV2	Non-FFS	25	3	87.50%	10.71%	14.28%
		FFS	4	24			
	CV3	Non-FFS	25	3	85.71%	10.71%	17.85%
		FFS	5	23			

		Average	25	3	85.71%	10.71%	17.85%
			5	23			

**Table 4:** Logistic regression cross-validation results.

Logistic regression model			Predict value		Hit ratio	Type I error	Type II error
			Non-FFS	FFS			
Actual value	CV1	Non-FFS	25	3	80.36%	10.71%	28.57%
		FFS	8	20			
	CV2	Non-FFS	26	2	82.14%	7.14%	28.57%
		FFS	8	20			
	CV3	Non-FFS	25	3	80.36%	10.71%	28.57%
		FFS	8	20			

	Average	Non-FFS	25	3	80.95%	9.52%	28.57%
		FFS	8	20			

**Table 5:** SVM cross-validation results.

SVM model			Predict value		Hit ratio	Type I error	Type II error
			Non-FFS	FFS			
Actual value	CV1	Non-FFS	26	2	73.21%	7.14%	46.42%
		FFS	13	15			
	CV2	Non-FFS	26	2	71.43%	7.14%	50.00%
		FFS	14	14			
	CV3	Non-FFS	26	2	71.43%	7.14%	50.00%
		FFS	14	14			

	Average	Non-FFS	26	2	72.02%	7.14%	48.81%
		FFS	14	14			

**Table 6:** Summary of classification results.

Model	Type I error	Type II error	Hit ratio	Ranking
Logistic regression	9.52%	28.57%	80.95%	2
SVM	7.14%	48.81%	72.02%	3
DT C5.0	10.71%	17.85%	85.71%	1

**Table 7:** Paired-samples *t* test.

Model	*t*-value	DF	Significant (two-tailed)
C5.0—logistic	−5.201	2	0.35
Logistic—SVM	−16.958	2	0.03
SVM—C5.0	9.823	2	0.10

**Table 8:** Selection of the research variables.

Variable classification	Variable code	Variable description and computation
Financial variables	*X*1	Accounts receivables/total assets
	*X*2	Gross profit/total assets
	*X*3	Inventory/current assets
	*X*4	Inventory/total assets
	*X*5	Net profit after tax/total assets
	*X*6	Net profit after tax/fixed assets
	*X*7	Cash/total assets
	*X*8	Log total assets
	*X*9	Log total liabilities
	*X*10	Interest protection multiples (debt service coverage ratio, times interest earned)
	*X*11	Gross profit margin
	*X*12	Operating expense ratio
	*X*13	Debt ratio
	*X*14	Inventory turnover
	*X*15	Cash flow ratio
	*X*16	Net profit ratio before tax
	*X*17	Accounts payable turnover
	*X*18	Revenue growth rate
	*X*19	Debt/equity ratio
	*X*20	Earnings before interest, taxes, depreciation, and amortization
	*X*21	Current liabilities/total assets
	*X*22	Total assets turnover
	*X*23	Account receivable/last year accounts receivable >1.1
	*X*24	Operation profit/last year operation profit >1.1

Nonfinancial variables	*X*25	Shareholding ratio of the major shareholders
	*X*26	Shareholding ratio of directors and supervisors
	*X*27	Whether the chairman concurrently holds the position of CEO
	*X*28	Board size
	*X*29	Pledge ratio of shares of the directors and supervisors

## Appendix

See Table 8.
